# Supplementary material for: Citrullinated fibrinogen-SAAs complex causes vascular metastagenesis
Source: Nat Commun. 2023 Aug 24;14:4960. doi: 10.1038/s41467-023-40371-1 (PMC10449786; doi:10.1038/s41467-023-40371-1)
Supplement: Supplementary file 1 — Supplementary Information File [file 41467_2023_40371_MOESM1_ESM.pdf]

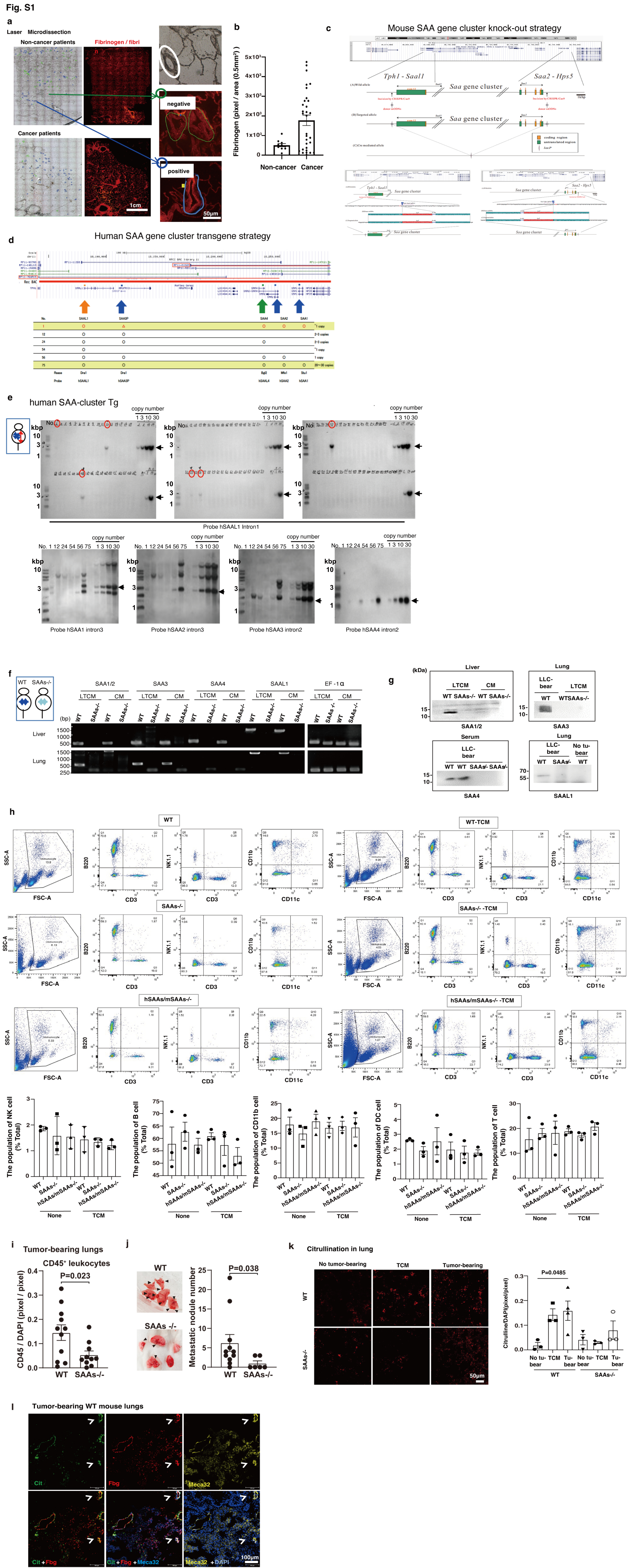
Fig. S1
Laser Microdissection
Non-cancer patients
Fibrinogen / fibri
Cancer patients
negative
positive
1cm
50μm
Fibrinogen (pixel / area (0.5mm²))
Non-cancer
Cancer
Mouse SAA gene cluster knock-out strategy
Tph1 - Saal1
Saa gene cluster
Saa2 - Hps5
Human SAA gene cluster transgene strategy
human SAA-cluster Tg
copy number
Probe hSAAL1 Intron1
Probe hSAA1 intron3
Probe hSAA2 intron3
Probe hSAA3 intron2
Probe hSAA4 intron2
SAA1/2
SAA3
SAA4
SAAL1
EF -1α
LTCM
CM
Liver
Lung
Serum
LLC-bear
No tu-bear
WT
SAAs-/-
hSAAs/mSAAs-/-
WT-TCM
SAAs-/- -TCM
hSAAs/mSAAs-/- -TCM
FSC-A
SSC-A
B220
NK1.1
CD11b
CD3
CD11c
The population of NK cell (% Total)
The population of B cell (% Total)
The population of CD11b cell (% Total)
The population of DC cell (% Total)
The population of T cell (% Total)
None
TCM
Tumor-bearing lungs
CD45⁺ leukocytes
P=0.023
CD45 / DAPI (pixel / pixel)
P=0.038
Metastatic nodule number
Citrullination in lung
No tumor-bearing
Tumor-bearing
P=0.0485
Citrulline/DAPI(pixel/pixel)
Tumor-bearing WT mouse lungs
Cit
Fbg
Meca32
Cit + Fbg
Cit + Fbg + Meca32
Meca32 + DAPI
100μm

Fig. S1

Replacement of mouse *Saa* cluster genes with human *SAA* cluster genes *in vivo*.

a) Non-stained (upper), Fbg-positive (blue circle), and negative (green circle) pulmonary small vessels derived from non-cancer and cancer patients. Scale bars, 1 cm (right), and 50 μm (left). **b)** Quantification of Fbg signals in lung autopsy samples shown in Fig. 1a. (n = 10 non-cancer patients, n = 31 cancer patients) **c)** Strategy to delete 70 kb of mouse *Saa* cluster genes. **d)** Transgene strategy to integrate ~200 kb of human *SAA* cluster genes. In three mouse lines, the presence of a full-length human *SAAs* region was confirmed by southern blot for each SAA probe (arrow). **e)** Genomic southern blot analysis for human *SAA* genes in human *SAA* cluster transgenic (Tg) mice. The signals of 1–30 copy numbers by the hSAAL1 probe were shown to validate the copy number of the human transgene in each mouse (red circle, upper panel). Mice possessing positive hSAAL1 signals were confirmed by the other probes for hSAA1, hSAA2, hSAA3, and hSAA4. Arrows and arrowheads indicate copy number control (lower). **f**) mRNA expression of *SAA* cluster genes in LTCM-stimulated and LLC-bearing mouse lungs and liver using RT-PCR analysis. SAA1 PCR amplified SAA1 and 2 due to a high homology of their sequences. CM and LTCM were prepared from the conditioned medium devoid of tumor cells or LLC cells, respectively. EF-1α is an endogenous control. Three independent experiments. **g)** Induction of SAAs in LTCM-stimulated and LLC-bearing mice detected by western blot. **h)** Flow cytometric analysis of DC, NK, CD11b$^+$, B and T cells in PBMC derived from normal and TCM-stimulating condition (n = 3). Gating strategy (upper) and each cell population (lower). **i)** Increased CD45$^+$ leukocytes in tumor-bearing-lungs from wild-type compared with those from SAAs-/- mice (n = 11 WT, n = 10 SAAs-/-). Student’s two-sided t-test. **j**) Representative photo of spontaneous metastasis of 3LL tumor, a spontaneous metastatic cell line in Lewis lung carcinoma (LLC), to the lungs (left, arrow indicates metastatic nodules) and lung metastasis number (right) (n = 11 WT, n = 6 SAAs-/- mice). Student’s two-sided t-test. **k)** IHC photo of citrullination in no tumor-bearing-, TCM-stimulated-, and tumor-bearing- wild-type and SAAs-/- mouse lungs (left). Quantification of the citrullination signals (right). (n = 3 WT; n = 3 SAAs-/-). One-way ANOVA with Bonferroni correction. **l)** Representative staining of citrullination, Fbg, and endothelial marker, MECA32, in a low magnified image related to Fig. 1i. Arrowheads indicate merged signals in small vessels/capillaries in tumor-bearing wild-type mouse lungs. Scale bars, 100 μm. Five independent experiments. In all graphs, data are mean ± SEM.

Fig. S2

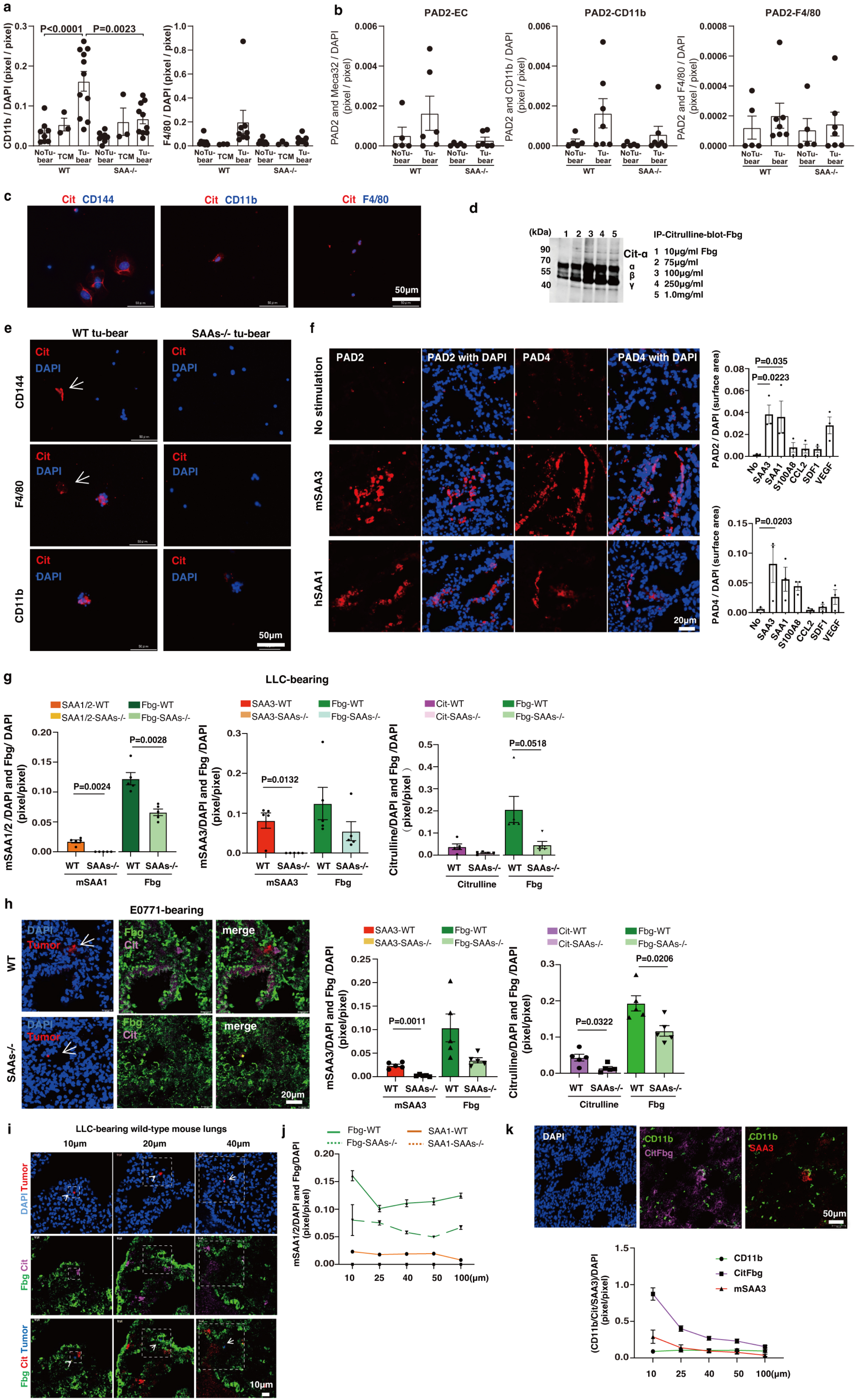

a
CD11b / DAPI (pixel / pixel)
P<0.0001
P=0.0023
NoTu-bear TCM Tu-bear NoTu-bear TCM Tu-bear
WT SAA-/-
F4/80 / DAPI (pixel / pixel)
b
PAD2-EC
PAD2-CD11b
PAD2-F4/80
PAD2 and Meca32 / DAPI (pixel / pixel)
PAD2 and CD11b / DAPI (pixel / pixel)
PAD2 and F4/80 / DAPI (pixel / pixel)
c
Cit CD144
Cit CD11b
Cit F4/80
50μm
d
IP-Citrulline-blot-Fbg
(kDa) 1 2 3 4 5
90 70 55 40
Cit-α
α
β
γ
1 10μg/ml Fbg
2 75μg/ml
3 100μg/ml
4 250μg/ml
5 1.0mg/ml
e
WT tu-bear
SAAs-/- tu-bear
CD144
F4/80
CD11b
Cit
DAPI
50μm
f
PAD2
PAD2 with DAPI
PAD4
PAD4 with DAPI
No stimulation
mSAA3
hSAA1
20μm
PAD2 / DAPI (surface area)
P=0.035
P=0.0223
PAD4 / DAPI (surface area)
P=0.0203
No SAA3 SAA1 S100A8 CCL2 SDF1 VEGF
g
LLC-bearing
SAA1/2-WT
SAA1/2-SAAs-/-
Fbg-WT
Fbg-SAAs-/-
mSAA1/2 /DAPI and Fbg/ DAPI (pixel/pixel)
P=0.0024
P=0.0028
WT SAAs-/- WT SAAs-/-
mSAA1 Fbg
SAA3-WT
SAA3-SAAs-/-
mSAA3/DAPI and Fbg /DAPI (pixel/pixel)
P=0.0132
mSAA3 Fbg
Cit-WT
Cit-SAAs-/-
Citrulline/DAPI and Fbg /DAPI (pixel/pixel)
P=0.0518
Citrulline Fbg
h
E0771-bearing
DAPI
Tumor
Fbg
Cit
merge
WT
SAAs-/-
20μm
P=0.0011
P=0.0322
P=0.0206
i
LLC-bearing wild-type mouse lungs
10μm 20μm 40μm
DAPI Tumor
Fbg Cit
Fbg Cit Tumor
10μm
j
Fbg-WT
Fbg-SAAs-/-
SAA1-WT
SAA1-SAAs-/-
mSAA1/2/DAPI and Fbg/DAPI (pixel/pixel)
10 25 40 50 100(μm)
k
DAPI
CD11b
CitFbg
CD11b
SAA3
50μm
(CD11b/Cit/SAA3)/DAPI (pixel/pixel)
CD11b
CitFbg
mSAA3
10 25 40 50 100(μm)

Fig. S2

Endothelial cells (EC)-mediated SAAs-CitFbg around metastatic tumor cells.

a) The number of $CD11b^+$ cells (n = 8 WT-no tumor; n = 3 WT-TCM; n = 11 WT- LLC tumor, n = 8 SAAs-/--no tumor; n = 3 SAAs-/- TCM; n = 10 SAAs-/--LLC tumor) and $F4/80^+$ cells (n = 8 WT-no tumor; n = 3 WT-TCM; n = 8 WT- LLC tumor, n = 8 SAAs-/--no tumor; n = 3 SAAs-/- TCM; n = 8 SAAs-/--LLC tumor) in tumor-bearing lung tissues in IHC analysis. One-way ANOVA with Bonferroni correction. **b)** Quantifications of PAD2 in EC (PAD2-EC) (n = 5 WT-no tumor; n = 6 WT-LLC tumor; n = 5 SAAs-/--no tumor; n = 6 SAAs-/--LLC tumor), $CD11b^+$ cells (PAD2-CD11b) (n = 5 WT-no tumor; n = 7 WT-LLC tumor; n = 5 SAAs-/--no tumor; n = 7 SAAs-/--LLC tumor) and $F4/80^+$ cells (PAD2-F4/80) (n = 5 WT-no tumor; n = 7 WT-LLC tumor; n = 5 SAAs-/--no tumor; n = 7 SAAs-/--LLC tumor) in no tumor-bearing and LLC-bearing lung tissues in IHC analysis. **c)** Citrullination signals on $CD144^+$ ECs, $CD11b^+$ cells, and $F4/80^+$ cells derived from LLC-bearing mouse lungs. Scale bars, 50 µm. Four independent experiments. **d**) Measurement of enzymatic activity of citrullination for various concentrations of Fbg. The citrullinated Fbg was detected after applying lung cells derived from tumor-bearing mice in western blot. Two independent experiments. **e)** Lung-derived cells citrullinate pre-coated Fbg. Three independent experiments. Arrows indicate CitFbg. Scale bar, 50 µm. **f)** PADs in mouse lung organ culture after application of mSAA3 and hSAA1 (left). Quantification of PAD signals 72 h after stimulation by various factors (right). Scale bars, 20 µm. **g)** Quantitative IHC signals of SAA1/2, SAA3, and citrulline under increased Fbg in the post-metastatic phase using LLC-bearing wild-type and SAAs-/- mouse lungs (n = 5 WT; n = 5 SAAs-/-). Student's two-sided t-test. **h)** Fbg and citrulline around metastatic tumor cells (arrow) in E0771-bearing wild-type and SAAs-/- mouse lungs (left). Scale bars, 20 µm. Quantitative signals of SAA3 and citrulline with Fbg staining in E0771-bearing wild-type and SAAs-/- lungs (right) (n = 5 WT; n = 5 SAAs-/-). Student's two-sided t-test. **i)** Calculation methods of IHC signals. Labeled metastatic cell was located at the central portion (arrowheads). Scale bars, 10 µm. **j**) Distance kinetics between tumor cells and proteins, such as SAA1/2 and Fbg, in LLC-bearing wild-type and SAAs-/- mouse lungs (n = 5 WT; n = 5 SAAs-/-). **k)** Representative IHC image of CD11b, CitFbg and SAA3 in lungs from LLC-bearing mice. Distance between SAA3-CitFbg and CD11b signals (lower) (n = 5). Scale bars, 50 µm. In all graphs, data are mean ± SEM.

Fig. S3

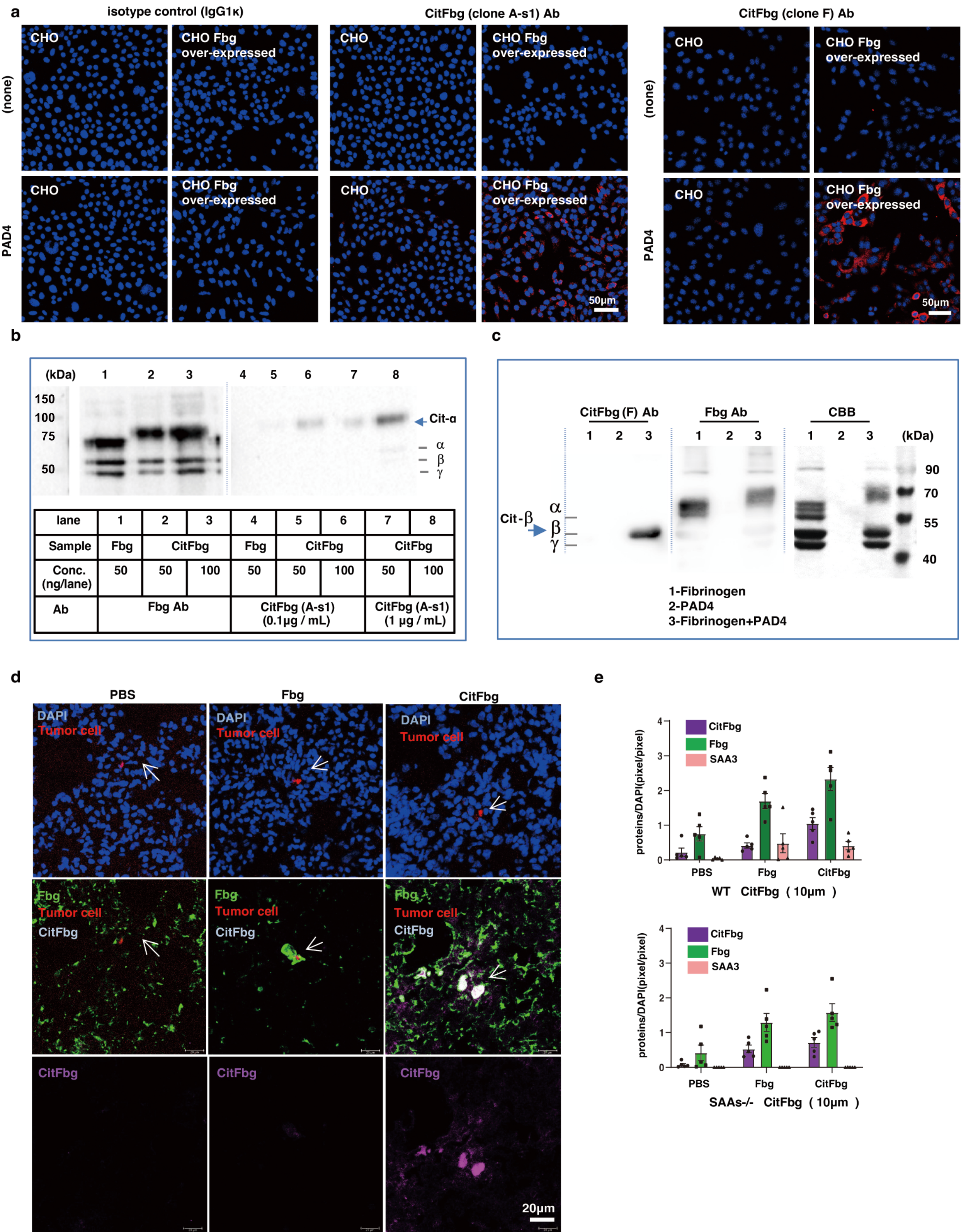

lane	1	2	3	4	5	6	7	8
Sample	Fbg	CitFbg		Fbg	CitFbg		CitFbg	
Conc. (ng/lane)	50	50	100	50	50	100	50	100
Ab	Fbg Ab			CitFbg (A-s1) (0.1μg / mL)			CitFbg (A-s1) (1 μg / mL)	

Fig. S3

Validation of anti-hCitFbg antibody and measurement of distance between CitFbg and tumor cells by the antibody.

a) Evaluation of antibodies for hCitFbg using hFbg-overexpressed CHO cells. The cells were citrullinated by PAD4 before IHC analysis. Scale bars, 50 µm. Two independent experiments. **b)** and **c)** specificity of antibodies for hCitFbg protein in western blot analysis. The bands of α and β chains of hCitFbg were detected by clone A-s1 (**b**) and clone F (**c**), respectively. Various combinations were demonstrated to determine the specificity and sensitivity of the A-s1 antibody. Two independent experiments. **d)** Immunohistochemical detection of injected hCitFbg with colocalization of tumor cells (arrow) using an anti-hCitFbg antibody (clone A-s1). Scale bars, 20 µm. Four independent experiments. **e)** Measurement of several protein signals around metastatic tumor cells in the pre-inoculation of PBS, Fbg, and CitFbg into wild-type mice. The calculation area was within 10 µm of tumor cells (n = 5 WT; n = 5 SAAs-/-). One-way ANOVA with Bonferroni correction. Data are mean ± SEM.

Fig. S4

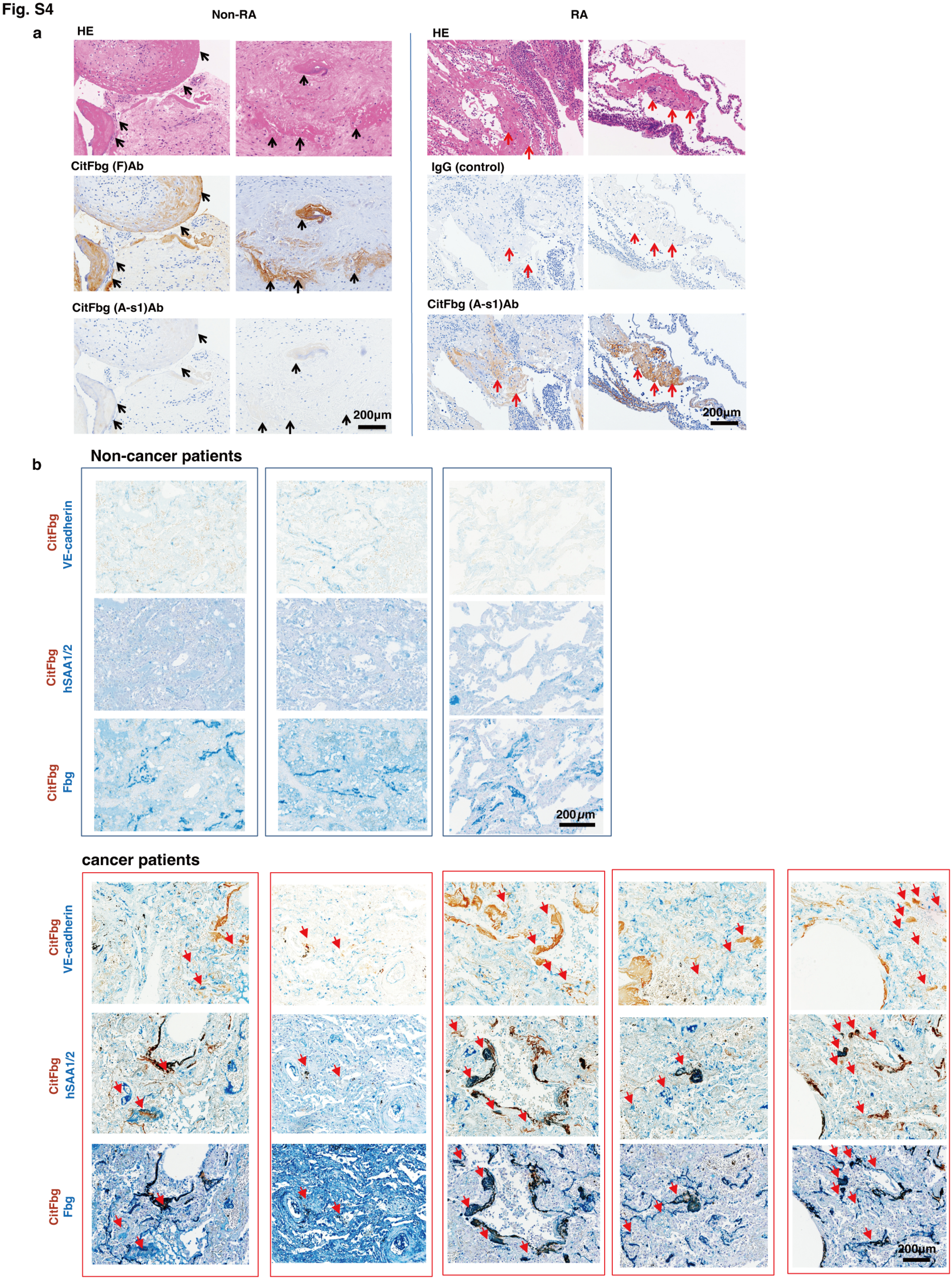
a
Non-RA
RA
HE
CitFbg (F)Ab
CitFbg (A-s1)Ab
IgG (control)
200μm
b
Non-cancer patients
cancer patients
CitFbg
VE-cadherin
CitFbg
hSAA1/2
CitFbg
Fbg
200μm

Fig. S4

The deposition of SAA1/2-CitFbg in pulmonary vessels in the lungs of patients.

a) IHC analysis for Fbg in joint tissues derived from RA and non-RA patients using clone A-s1 and clone F antibodies. An anti-CitFbg Clone A-s1 antibody reacted to CitFbg (red arrow) in RA but not Fbg (black arrow) of non-RA, specifically IHC detection for CitFbg. Scale bars, 200 μm. Four independent experiments. **b**) Representative images with double staining of CitFbg/VE-cadherin and CitFbg/SAA1/2 and CitFbg/Fbg in lung specimens derived from non-cancer patients (n = 3) and cancer patients (n = 3) (data related to Fig. 4c). Arrows indicate the same position in continuous sections. Scale bars, 200 μm.

Fig. S5

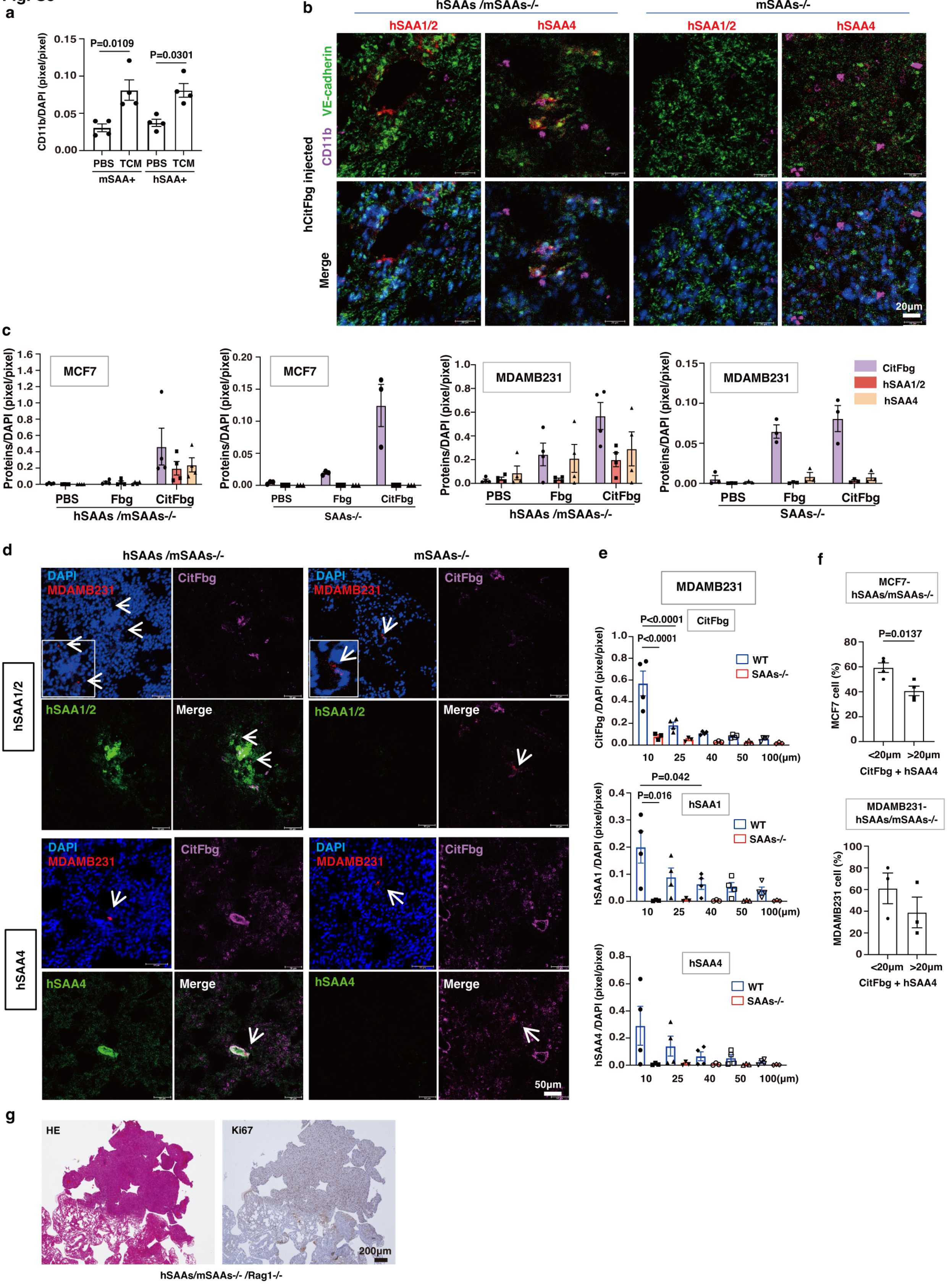

a
CD11b/DAPI (pixel/pixel)
P=0.0109
P=0.0301
PBS TCM PBS TCM
mSAA+
hSAA+
b
hSAAs /mSAAs-/-
mSAAs-/-
hSAA1/2
hSAA4
hSAA1/2
hSAA4
hCitFbg injected
CD11b VE-cadherin
Merge
20μm
c
MCF7
MCF7
MDAMB231
MDAMB231
Proteins/DAPI (pixel/pixel)
PBS Fbg CitFbg
hSAAs /mSAAs-/-
SAAs-/-
CitFbg
hSAA1/2
hSAA4
d
hSAAs /mSAAs-/-
mSAAs-/-
hSAA1/2
hSAA4
DAPI
MDAMB231
CitFbg
hSAA1/2
hSAA4
Merge
50μm
e
MDAMB231
CitFbg
P<0.0001
P<0.0001
CitFbg /DAPI (pixel/pixel)
WT
SAAs-/-
10 25 40 50 100(μm)
hSAA1
P=0.042
P=0.016
hSAA1 /DAPI (pixel/pixel)
hSAA4
hSAA4 /DAPI (pixel/pixel)
f
MCF7-
hSAAs/mSAAs-/-
P=0.0137
MCF7 cell (%)
<20μm >20μm
CitFbg + hSAA4
MDAMB231-
hSAAs/mSAAs-/-
MDAMB231 cell (%)
g
HE
Ki67
200μm
hSAAs/mSAAs-/- /Rag1-/-

Fig. S5

The induction of the protein complex of SAA-CitFbg is an initiator for cancer metastasis in SAA-humanized mice.

a) Quantifications of $CD11b^{+}$ cells in the lungs from WT (mSAA+) or hSAAs/mSAAs-/- (hSAA+) mice with or without TCM-injection (n = 4). One-way ANOVA with Bonferroni correction. **b)** Representative photos of the induction of hSAA1/2 and hSAA4 by injecting hCitFbg to hSAAs/mSAAs-/- mice. Scale bars, 20 μm. **c)** Measurement of signals of the hSAA1/2, hSAA4, and hCitFbg after injecting PBS, hFbg, and hCitFbg into hSAAs/mSAAs-/- and mSAAs-/- mice that were followed by tumor cell injection. The CitFbg signals were kept in both genotypes (n = 4 hSAAs/mSAAs-/-; n = 3 mSAAs-/-) One-way ANOVA with Bonferroni correction. **d)** Representative photos of metastatic MDAMB231 cells (arrows, magnified lower left) trapped by the hSAAs-hCitFbg complex. Scale bars, 50 μm. **e)** Distances among hCitFbg, hSAA1, hSAA4, and MDAMB231 cells shown in (**d)** were analyzed. **d** and **e** are related to Fig. 5d, e**)** (n = 4 hSAAs/mSAAs-/-; n = 3 mSAAs-/-). One-way ANOVA with Bonferroni correction. **f)** The percentage of MCF7 and MDAMB231 cells located within 20 μm from hSAA4-hCitFbg (n = 4 hSAAs/mSAAs-/- for MCF7; n = 4 hSAAs/mSAAs-/- for MDAMB231). Student's two-sided t-test. **g)** (Related to Fig. 5g) Representative HE and Ki67 stainings of lungs 3 weeks after injection of tumor cells in hSAAs/mSAAs-/-/Rag1-/- and mSAAs-/-/Rag1-/- mice. Scale bars, 200 μm. In all graphs, data are mean ± SEM.

Fig. S6

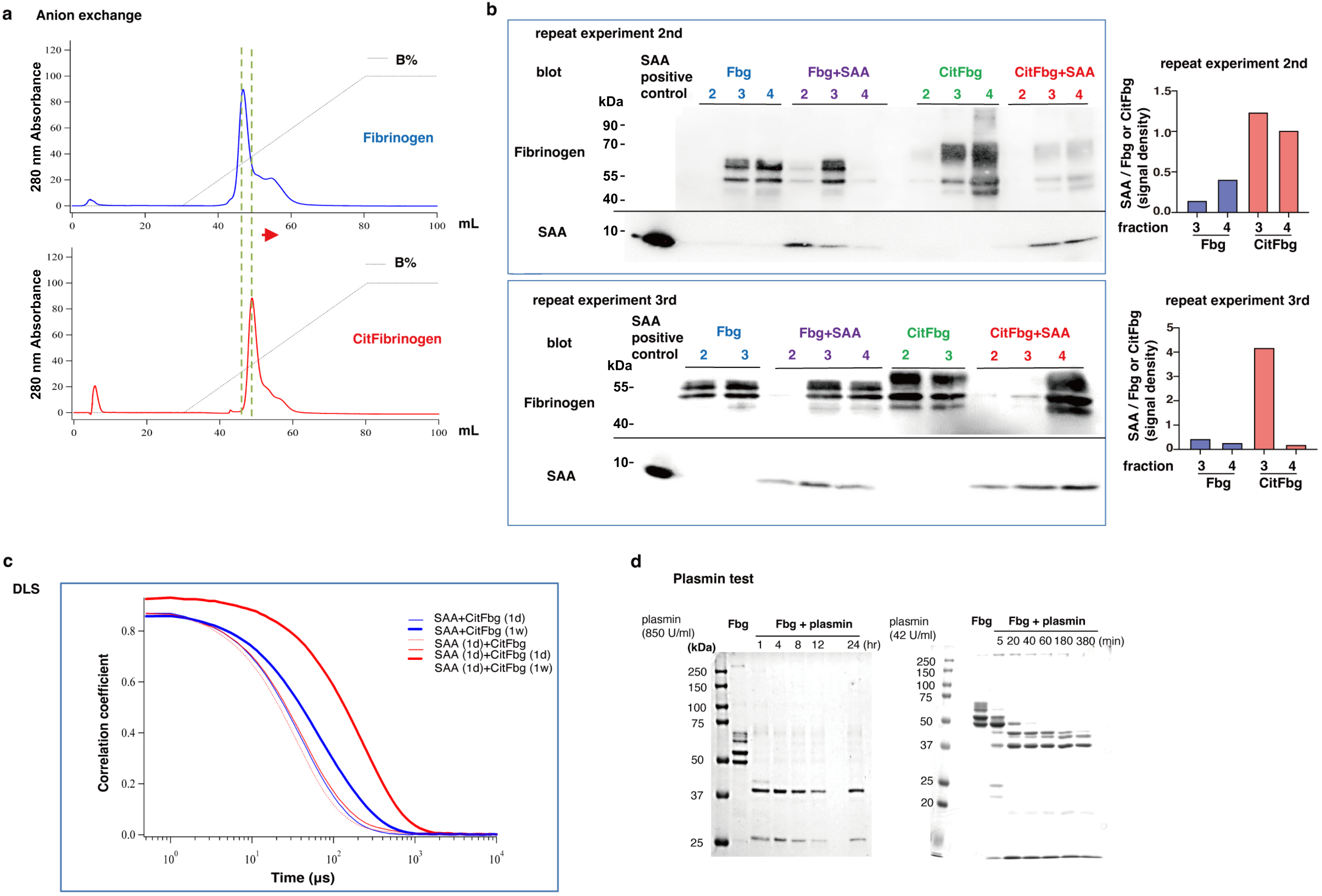
a
Anion exchange
280 nm Absorbance
B%
Fibrinogen
CitFibrinogen
mL
b
repeat experiment 2nd
blot
SAA positive control
Fbg
Fbg+SAA
CitFbg
CitFbg+SAA
kDa
Fibrinogen
SAA
SAA / Fbg or CitFbg (signal density)
fraction
repeat experiment 3rd
c
DLS
SAA+CitFbg (1d)
SAA+CitFbg (1w)
SAA (1d)+CitFbg
SAA (1d)+CitFbg (1d)
SAA (1d)+CitFbg (1w)
Correlation coefficient
Time (μs)
d
Plasmin test
plasmin (850 U/ml)
(kDa)
Fbg
Fbg + plasmin
(hr)
plasmin (42 U/ml)
(min)

Fig. S6

Characterization of hFbg and hCitFbg in the presence and absence of SAA.

a) hFbg and hCitFbg were analyzed using anion exchange chromatography. The elution of CitFbg occurred at a higher NaCl concentration than that of Fbg, consistent with converting the positively charged guanidino group to the neutral ureido group by citrullination. Three independent experiments. **b**) Examination of SAA1 associated with Fbg and CitFbg based on gel filtration chromatography (Superdex 200 Increase) and western blotting analysis. The experiments shown in Fig. 6b were repeated an additional two times. CitFbg bound more SAA1 than Fbg. **c)** DLS correlation curves. (1) SAA1 and CitFbg co-incubation for 1 day (blue), (2) SAA1 and CitFbg co-incubation for 1 week (blue solid line), (3) SAA1 1 day incubation and addition of CitFbg (red dotted line), (4) SAA1 1 day incubation followed by co-incubation with CitFbg for additional 1 day (red line), and (5) SAA1 1 day incubation followed by co-incubation with CitFbg for 1 week (red solid line) were depicted. In the data acquisition, SAA1 (0.2 mg/mL) and CitFbg (1 mg/mL) were dissolved in 50 mM Hepes, pH 7.7 for sample (3) or in 50 mM Hepes, pH 7.7, 10 mM $MgCl_2$ for samples (1), (2), (4), and (5). All samples were incubated at 37°C. **d)** (related to Fig. 6e) Degradation assay of Fbg at two concentrations of plasmin. The gel was stained with CBB.

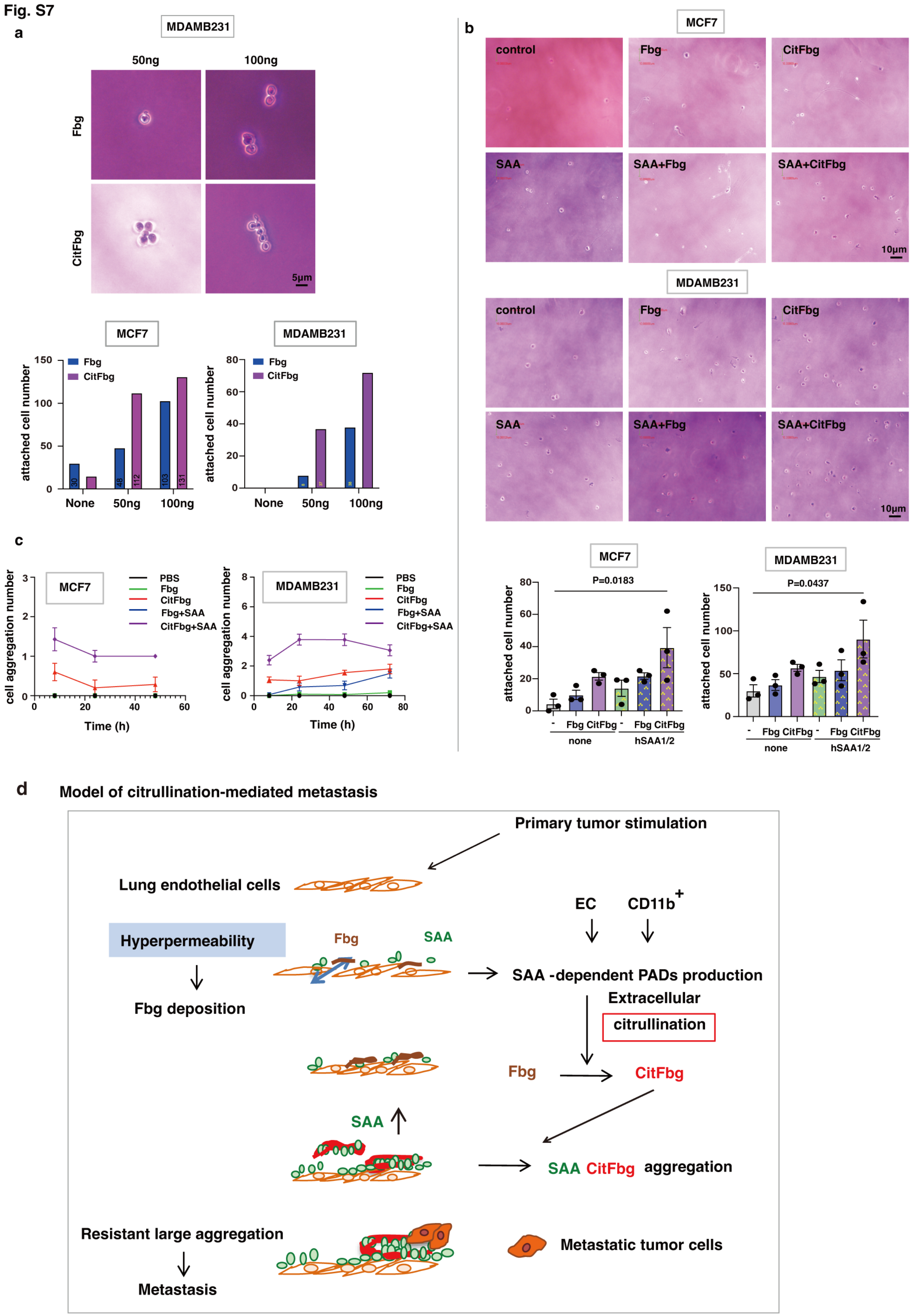

Fig. S7
a
MDAMB231
50ng
100ng
Fbg
CitFbg
5μm
MCF7
MDAMB231
attached cell number
Fbg
CitFbg
None
50ng
100ng
b
MCF7
control
Fbg
CitFbg
SAA
SAA+Fbg
SAA+CitFbg
10μm
MDAMB231
P=0.0183
P=0.0437
none
hSAA1/2
c
cell aggregation number
PBS
Fbg
CitFbg
Fbg+SAA
CitFbg+SAA
Time (h)
d
Model of citrullination-mediated metastasis
Primary tumor stimulation
Lung endothelial cells
EC
CD11b+
Hyperpermeability
Fbg
SAA
SAA -dependent PADs production
Fbg deposition
Extracellular
citrullination
CitFbg
SAA CitFbg aggregation
Resistant large aggregation
Metastatic tumor cells
Metastasis

Fig. S7

Aggregation of SAA-CitFbg protein complex and tumor cells

a and **b**) Human tumor cell attachment assay to Fbg-, CitFbg-, and SAA1-CitFbg- coated plates 1 h after cell seeding. Representative photo (left in **a**). The number of MCF7 and MDAMB231 cells exposed to various concentrations of Fbg and CitFbg-coating plates (right in **a** Scale bars, 5 μm). Attached are MCF7 and MDAMB231 cells to SAA1-CitFbg-coating plates (upper in **b** Scale bars, 10 μm) and the cell numbers of attached tumor cells (lower in **b**) ($n = 3$). One-way ANOVA with Bonferroni correction. (**c**) Time course of cell aggregation of MCF7 and MDAMB231 cells (5 or more cells in aggregation) cultured on various protein complex-coating plates. ($n = 3$ for MCF7 and $n = 4$ for MDAMB231 cells). Data are mean ± SEM. **d)** Model of citrullination-mediated metastasis. SAAs induced extracellular citrullination of Fbg in hyperpermeable spots. The citrullination on the pulmonary ECs was catalyzed by PADs. Finally, SAAs-CitFbg aggregation recruits circulating tumor cells.

Fig.S8 Full gel and Image

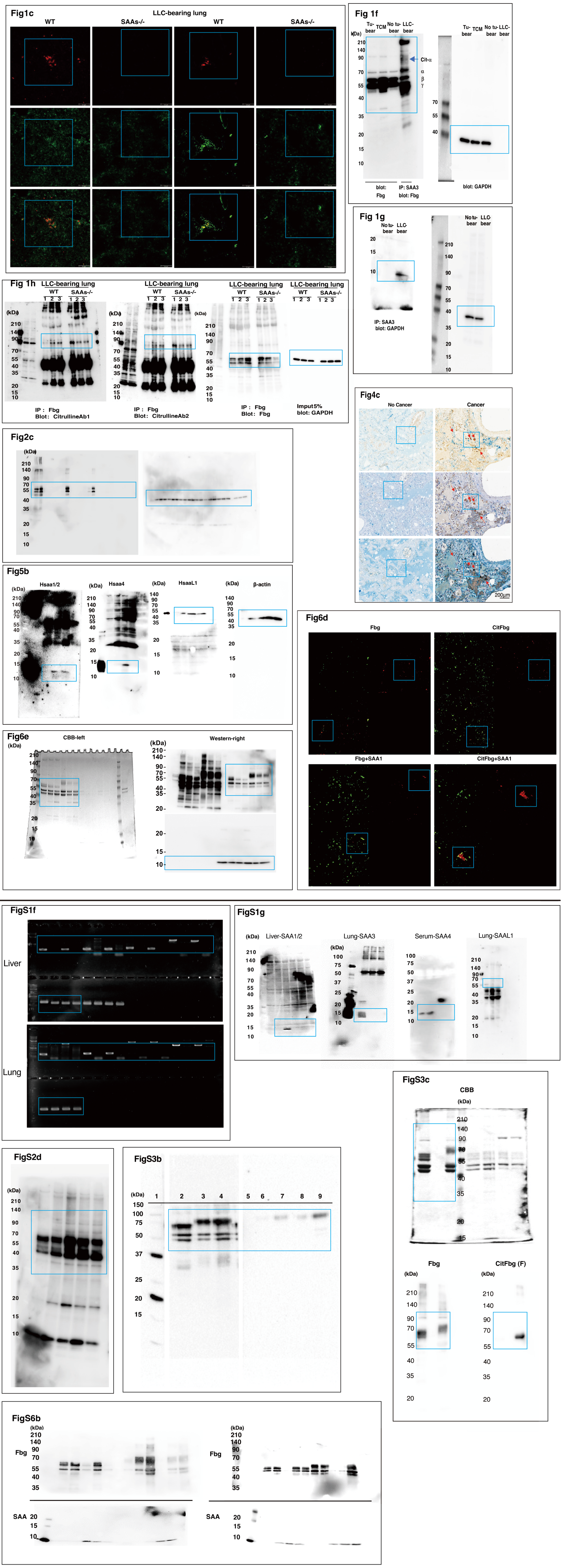

Fig. S8

Full gels for all Western blot images used for data presentation in Figs. 1f, 1g, 1h, 2c, 5b, 6e, S1g, S2d, S3b, S3c, and S6b, and original pictures used for data presentation in Figs. 1c, 4c, and 6d.

Supplementary Table 1

Gene Symbol	Gene Title	Non-cancer patients (Raw)	Cancer Patients (Raw)	Fold change	RefSeq Transcript ID
RBMS1	RNA binding motif, single stranded interacting protein 1	5.5950274	433.03644	5.755966	NM_002897///NM_016836///NM_016839
RPL6	ribosomal protein L6	4.358773	328.1133	5.715895	NM_000970///NM_001024662
RPL10A	ribosomal protein L10a	5.5631275	324.13657	5.346328	NM_007104
MORF4L1	mortality factor 4 like 1	4.7582364	264.61035	5.279065	NM_001265603///NM_001265604///NM_001265605///NM_006791///NM_206839
RPS4X	ribosomal protein S4, X-linked	6.559085	355.10107	5.240358	NM_001007
**SAA1///SAA2///SAA2-SAA4**	**serum amyloid A1///serum amyloid A2///SAA2-SAA4 readthrough**	31.830032	1678.5461	5.202446	NM_000331///NM_001127380///NM_001178006///NM_001199744///NM_030754///NM_199161
GPX3	glutathione peroxidase 3 (plasma)	7.483164	361.4908	5.075933	NM_002084
RPS10	ribosomal protein S10	10.147761	415.12775	4.836088	NM_001014///NM_001203245///NM_001204091
RPL23A///SNORD42A	ribosomal protein L23a///small nucleolar RNA, C/D box 42A	6.979831	259.7433	4.699517	NM_000984///NR_000014
HUWE1	HECT, UBA and WWE domain containing 1, E3 ubiquitin protein ligase	10.953302	396.56732	4.659895	NM_031407
NPM1	nucleophosmin (nucleolar phosphoprotein B23, numatrin)	9.6121855	343.58865	4.641440	NM_001037738///NM_002520///NM_199185
RPL24	ribosomal protein L24	19.095425	682.21045	4.640684	NM_000986
RPS3A///SNORD73A	ribosomal protein S3A///small nucleolar RNA, C/D box 73A	6.628325	212.60031	4.485122	NM_001006///NM_001267699///NR_000007
ACTG1	actin, gamma 1	4.8529983	152.97282	4.460021	NM_001199954///NM_001614///NR_037688
MAP2K5	mitogen-activated protein kinase kinase 5	44.27736	1391.8768	4.456085	NM_001206804///NM_002757///NM_145160///NM_145161///NM_145162
LDHB	lactate dehydrogenase B	5.4338665	169.75435	4.447092	NM_001174097///NM_002300
RPS17///RPS17L	ribosomal protein S17///ribosomal protein S17-like	5.9380016	175.185	4.364524	NM_001021///NM_001199057
RPS11///SNORD35B	ribosomal protein S11///small nucleolar RNA, C/D box 35B	19.39767	535.12146	4.267677	NM_001015///NR_001285
RPL21///RPL21P28///SNORA27///SNORD102	ribosomal protein L21///ribosomal protein L21 pseudogene 28///small nucleolar RNA, H/ACA box 27///small nucleolar RNA, C/D box 102	11.757041	309.21115	4.198762	NM_000982///NR_002574///NR_002575///NR_026911
KCMF1	potassium channel modulatory factor 1	20.280977	504.82816	4.119360	NM_020122
HMGB1	high mobility group box 1	10.717781	261.31653	4.089487	NM_002128
RPL38	ribosomal protein L38	13.629604	328.38217	4.072327	NM_000999///NM_001035258

Supplementary Table 1

The gene list related to the pre-metastatic phase using Fbg-deposited pulmonary vessels from noncancer and cancer patients. Microarray data up-regulated over 4-fold in cancer patients (No. 4) as compared to noncancer patients (No. 2) are shown. Related to Fig. 1a and b.

Supplementary Table 2

patients	non-cancer	number	cancer	number
	neurodegenerative disease	5	hepatocellular carcinoma	18
	aneurysm	4	pancreatic cancer	11
	infarction	4	gastric cancer	7
	cerebral hemorrhage	2	cholangiocarcinoma	5
	cardiomyopathy	2	esophageal cancer	5
	primary biliary cholangitis	2	ovarian cancer	4
	arrhythmia	2	colon cancer	3
	liver failure	2	melanoma	2
	liver cirrhosis	1	bladder cancer	2
	heart failure	1	gallbladder cancer	2
	respiratory failure	1	prostatic cancer	2
			malignant lymphoma	1
			adrenal cancer	1
			breast cancer	1
			duodenal papilla cancer	1
			pharyngeal cancer	1
			kidney cancer	1

Supplementary Table 2

A list for the samples derived from non-cancer patients and cancer patients. These were analyzed in Figs. 1, 4, and S4.
